# Update on Molecular Pathology of Cutaneous Melanocytic Lesions: What is New in Diagnosis and Molecular Testing for Treatment?

**DOI:** 10.3389/fmed.2014.00039

**Published:** 2014-10-31

**Authors:** Adriana C. H. van Engen-van Grunsven, Heidi Kusters-Vandevelde, Patricia J. T. A. Groenen, Willeke A. M. Blokx

**Affiliations:** ^1^Department of Pathology, Radboud University Medical Center, Nijmegen, Netherlands; ^2^Department of Pathology, Canisius Wilhelmina Hospital, Nijmegen, Netherlands

**Keywords:** review, Spitz, congenital nevus, melanoma, molecular pathology, targeted therapy

## Abstract

In this article, we give an update on recent findings regarding molecular pathology in cutaneous melanocytic tumors. The focus lies on use of genetics in the diagnosis of distinct subtypes of spitzoid tumors that are often characterized by specific phenotypic–genotypic alterations that can frequently be recognized by adequate histological examination. Typical illustrating cases are given in order to increase recognition of these lesions in daily dermatopathology practice. New molecular findings in the pathogenesis of congenital melanocytic tumors and neurocutaneous melanosis are reviewed. In addition, use of mutation analysis in the differential diagnosis of melanoma metastasis is discussed. Finally, application of mutation analysis in targeted therapy in advanced melanoma with advantages of new techniques such as next generation sequencing is described.

## Introduction

During the past 20 years, there has been a rapid development of molecular techniques increasing the possibilities of genetic testing in all kinds of tumors including melanocytic tumors. This has led to a rapid gain in our knowledge on the development of melanocytic tumors, which can help in diagnosis, prognosis, and treatment of melanocytic tumors. In 2010, we wrote a review on the molecular pathology of these tumors (1). Since developments in the field are fast, we will address in this paper relevant new findings from the recent years within the field of molecular pathology in melanocytic lesions.

The first part has a focus on new findings with respect to diagnosis and pathogenesis. The focus will be on new molecular findings in spitzoid tumors, in congenital melanocytic nevi (CMN) and neurocutaneous melanocytosis, and the use of molecular tests in the (differential) diagnosis of melanoma.

The second part will will be devoted to application of molecular pathology in the treatment of melanoma, and will briefly address new technological developments such as next generation sequencing (NGS) techniques in this setting.

## Part 1: Molecular Pathology in the Diagnosis and Pathogenesis of Melanocytic Tumors

**Figure 1:**
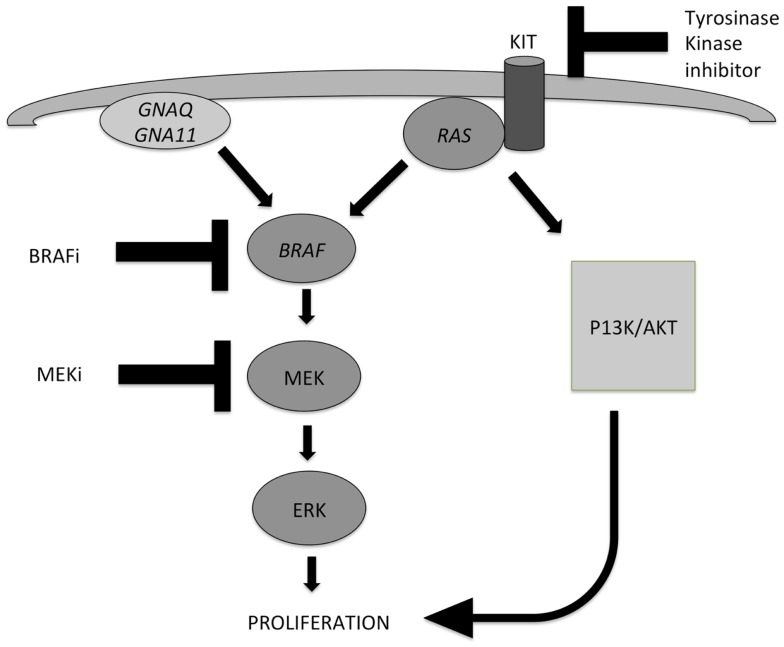
**Two important pathways involved in the development of melanocytic tumors and melanoma: the MAPK pathway and the AKT/PI3K-pathway**. Activation of both routes leads to proliferation. *NRAS* plays an important role in both pathways. Mutations in *GNAQ, GNA11*, and *BRAF* lead to activation of the MAPK pathway only, while mutations in *KIT* and *NRAS* can activate both pathways. Different inhibitors can be applied in targeted therapy of advanced melanoma patients, and their points of action are indicated also. MEK inhibitors can be effective in case of several different gene mutations in the MAPK pathway, because they exert their effect in the distal part of the pathway.

Early events in the development of melanocytic tumors are often hotspot mutations in genes involved in the MAPK pathway, which is one of the most important pathways involved in melanocytic tumor development (Figure 1). Important oncogenes in this pathway are *BRAF* (7q34), *NRAS* (1p13), *HRAS* (11p15), *GNAQ* (9p21), *GNA11* (19p13), and *KIT* (4q12*)* (2–5). Mutations in these genes are mostly mutually exclusive, by themselves do not cause malignant progression, stay present with malignant progression, and activate the MAPK pathway. Different subtypes of benign and malignant melanocytic tumors are characterized by different mutations in these genes of the MAPK pathway.

In common nevi for instance, *BRAF* and *NRAS* mutations are present in 60–87.5% (6, 7) and 20%, respectively. In large congenital nevi upto 80%, *NRAS* mutations are reported (7, 8). In blue nevi, mainly *GNAQ* (83%) and *GNA11* (7%) mutations are found (9), and in Spitz nevi, *HRAS* mutations are reported in 20–29% (7, 10).

Especially, in Spitz tumors, several new data indicate that these tumors are genetically more diverse than was previously thought. We will discuss these new findings below in part 1, together with new insights in the pathogenesis of CMN and the rare disease of neurocutaneous melanocytosis. We will also address the role of molecular pathology in the differential diagnosis of (metastatic) melanoma.

The distinct mutations in different melanoma types will be discussed later in part 2 (see also Table 1).

**Table 1:** **Overview of frequencies of gene mutations in different melanoma subtypes derived from different locations**.

Localization primary melanoma	*BRAF*	*NRAS*	*KIT*	*GNAQ*	*GNA11*
	*7q34*	*1p13.2*	*4q12*	*9p21*	*19p13*
Melanoma from CSDS/LMM	8%	15%	28%	1.4%	0
Melanoma from NCSD skin	60%	22%	0%-very low	0	0
ALM	22%	10%	23–36%	0	0
Mucosal melanoma	3–11%	5–24%	16–39%	0	0
Uvea melanoma	0%	0%	0%	45–50%	32%
Melanoma from the CNS	0%	0-low in adults. Frequently mutated in melanoma in context of NCM in children.	0%	30% (adults)	30% (adults)
Sensitive to treatment with	*BRAF* inhibitors	MEK inhibitors. Resistant to BRAFi	Imatinib, nilotinib, sunitinib, dasatinib	(Pre-clinical) MEK inhibitors	(Pre-clinical) MEK inhibitors

*In different studies, there is some variation in reported frequencies (5, 11, 12)*.

*CSDS, chronic sun damaged skin; LLM, lentigo malignant melanoma; ALM, acrolentiginous melanoma; CNS, central nervous system*.

### What is new in spitzoid melanocytic tumors?

At present, roughly three subgroups of spitzoid melanocytic tumors can be identified based on distinct genetic alterations. The first one is the group of the *HRAS*-mutated spitzoid tumors (13). The second group is the one of the *BAP1*-mutated “spitzoid lesions” (14, 15), and the third group consists of spitzoid tumors with kinase fusion (16).

The first two groups seem to be characterized mostly by a typical phenotype that can be recognized or at least suspected upon histological evaluation.

Most of the *HRAS*-mutated spitzoid tumors are typically wedge shaped, dermal-based lesions, with an infiltrative margin, consisting of mostly spindle-shaped cells, and showing marked desmoplasia (13, 17). This group is relevant to discriminate because of the favorable prognosis and to prevent melanoma overdiagnosis.

Several studies have reported the presence of *HRAS* mutations in spitzoid tumors with benign behavior, and absence in clear-cut spitzoid melanomas (10, 17, 18). There is only one recent paper mentioning the occurrence of *HRAS* mutations in upto 10% (2/20 cases examined) of primary cutaneous melanomas (19). In this paper, no histology of the lesions is shown or described; therefore, whether these lesions were spitzoid or not remains unclear, and no follow-up data of the patients are included to confirm the proposed diagnosis of melanoma by the authors. Furthermore, this paper also gives mutation frequencies of *BRAF* (25%) and *NRAS* (10%) that are quite different from most studies in the melanoma field.

**Figure 2:**
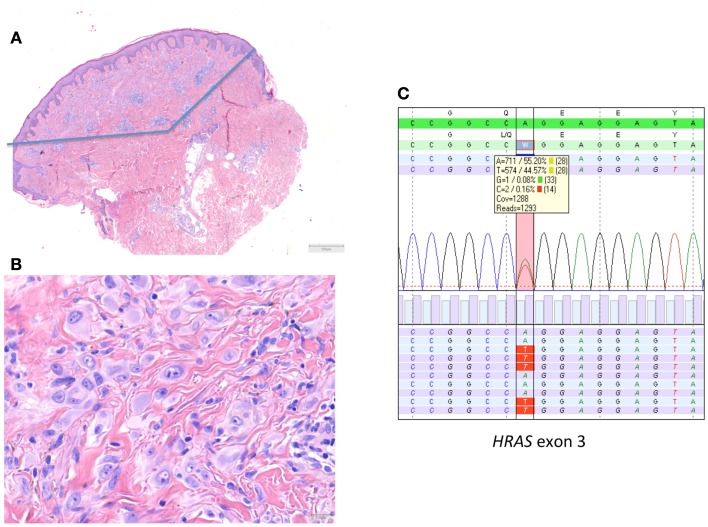
***HRAS*-mutated spitzoid tumor in a female patient aged 47 years**. The lesion was located on the right lower arm and measured 0.8 cm in diameter. The lesion was diagnosed elsewhere as STUMP (Spitzoid tumor of uncertain malignant potential). **(A,B)** The lesion was symmetrical, wedge shaped, mainly dermal located, with epithelioid spitzoid-looking cells in an eosinophilic hyalinized stroma [**(A)** HE 25×, **(B)** HE 400×]. There were 2 mitoses present per 1 mm^2^. Deep mitoses were absent. **(C)** Sequence plot of the mutation *HRAS* c.182A >T(p.(Gln61Leu)) with 44% of mutant alleles. The genomic sequence of the gene investigated is marked in green bars, and the protein sequence information is on top of the gene sequence. The combined (forward and reverse) gene sequence information is highlighted in light green, and the expected protein sequence is placed on top. The forward sequence information is indicated as light blue bars and reverse sequence information as purple bars. The square box shows the number and percentage of the different nucleotides at the variant position; the red vertical bar indicates the *HRAS* hotspot mutation position. The sequence plots are generated in SeqNext (from JSI Medical Systems GmbH). Note that the *HRAS* RefSeq is NM_005343.2.

In 2010, we described a series of 24 *HRAS*-mutated spitzoid melanocytic tumors (13). In 7/24 (29%) of these lesions, the initial diagnosis or important differential diagnosis had been melanoma based upon histological examination alone. These were mainly cases in adults that displayed rather frequent or deep mitotic activity. In five cases, more than 2 mitoses/1 mm^2^ or deep mitoses were present. In this series with a mean and median follow-up of 10.5 years, no recurrences or metastases occurred. An example of a *HRAS*-mutated spitzoid lesion is depicted in Figure 2.

The second group, which we preferentially call MBAITs (melanocytic *BAP1*-associated intradermal tumors, but are also called Wiesner tumor or BAPoma) are often polypoid, dermal based, consisting of large epitheloid “spitzoid-looking” cells, that can have a small common nevus component at the margin (especially in *BAP1*-germline mutated lesions), and in one-third of cases, there are prominent tumor-infiltrating lymphocytes (TILs).

These *BAP1*-mutated melanocytic lesions were first described by Wiesner et al. They described two families, one in Australia and one in Germany, in which a total of 16 individuals were affected by atypical cutaneous melanocytic tumors, in association with cutaneous and uvea melanomas (14). The affected family members were found to have a *BAP1*-germline mutation. Subsequently, it was found that these spitzoid-looking MBAITs, besides a *BAP1* mutation, also contained a *BRAF* mutation. Later, these lesions were also described in a sporadic setting in so-called atypical spitzoid tumors (ASTs), without having an underlying *BAP1-*germline mutation (15, 20). In 2012, Wiesner et al. described a series of 32 ASTs (20). Nine cases (28%) showed BAP 1 protein expression loss while BRAF protein expression was present. In 8/9 cases (89%), a BRAF^V600E^ mutation was found, and in 5/9 cases (55%), a somatic *BAP1* mutation was present. No *HRAS* mutations were found. Histology was comparable to the *BAP1*-germline mutation associated MBAITs, demonstrating a dermal-based lesion, plump epithelioid cells, giant cells, with in 1/3 cases prominent TILs, and absence of prominent fibrosis. These cases were not described to contain a small nevoid component in contrast to the germline *BAP1*-associated cases.

Yeh et al. recently described genomic loss, determined with array CGH, of >1 Mbp of chromosome 3 in a region containing the *BAP1* locus in a series of 29 cases out of 436 ambiguous melanocytic tumors (6.7%) (21): 22 cases showed partial loss of chromosome 3, while 7 cases demonstrated monosomy of chromosome 3. In 11 cases *BAP1* mutation analysis was performed with in 10 cases a loss of function mutation of *BAP1*, and in the remaining single case with wild-type *BAP1*, BAP1 protein expression was lost on the immunohistochemical level. In the cases with loss of 1 copy of *BAP1*, the BAP1 protein expression was always lost. So, immunohistochemistry for BAP1 protein seems to correlate well with the genetic findings. Reported follow-up in their series was good, although short (median 17 months), with no recurrences, and in one patient a negative sentinel lymph node. Morphology of 17 lesions with biallelic loss of *BAP1* that looked spitzoid was as described previously by others, with TILs being present in 50% of cases, and 31% having a small nevoid component at the margin. In these cases, a junctional component was present composed of the common nevus component. In one case without a common nevus component, a junctional spitzoid component was present. In 12/17 cases, a BRAF^V600E^ mutation was present, 4 cases were wildtype *BRAF*, and 1 case showed an NRAS^Q61R^ mutation. The latter is rarely reported at present, but we have also encountered such a case ourselves recently (paper submitted). In our patient, no underlying *BAP1* mutation was found. The other reported *NRAS-*mutated case (21) in literature was not tested for a germline mutation in *BAP1*. Most of the MBAIT cases reported thus far do not seem to be tested for the presence of *NRAS* mutations, so the number of *NRAS*-mutated cases may be larger, and the genetic make-up of these *BAP1*-associated melanocytic lesions could be broader than currently thought.

The fact that the combined MBAIT lesions show only *BAP1* loss in the epithelioid component suggests that they probably develop from a common nevus (that is mostly *BRAF* and seldom *NRAS* mutated) (7).

MBAITs probably have a low risk of developing into melanoma, but at present, data about behavior are insufficient to draw definite conclusions.

In the two largest series thus far by Pouryazdanparast et al. (22) and Yeh et al. (21), reporting 28 and 29 cases, respectively, follow-up was favorable without recurrences. Follow-up was relatively short with a mean of 21 months and median of 17 months, respectively. Pouryazdanparast performed FISH [using probes targeting chromosome 6p25 (RREB1), chromosome 6q23 (MYB), chromosome 11q13 (CCND1), and the centromeric portion of chromosome 6 (CEP 6)] on these lesions, which was negative in all cases.

The difference in outcome between the uveal lesions and the skin lesions with a *BAP1* mutation may be related to the presence of different oncogenic driver mutations in uveal lesions, which harbor *GNAQ* or *GNA11* mutations (23) instead of *BRAF* or *NRAS* mutations.

The suggested progression-promoting effect of mutated BAP1 is in line with the tumor suppressive function of intact BAP1 as a deubiquitylase required for efficient assembly of the homologous recombination (HR) factors BRCA1 and RAD51 after DNA double-strand breaks (DSBs) (24, 25). BAP1 is recruited to DNA damage sites together with ASXL1 and deubiquitilates Ub-H2AK119 at sites of DNA damage (24, 25). In this way, it promotes error-free repair of these lesions. Defective HR and increased sensitivity to radiation due to BAP1 deficiency may, therefore, lead to genomic instability, a hallmark of cancer (24, 25). Moreover, BAP1 prevents proteasomal degradation of the conserved epigenetic regulator host cell factor-1 (HCF-1) and, consistent with this, Dey et al. showed that BAP1 KO splenocytes contained far less HCF-1 than their wild-type counterparts (26). It is thought that BAP1 regulates gene expression via stabilization of HCF-1 (26). These two examined functions of BAP1 could explain tumor progression due to altered BAP1 expression.

The most important reason for recognition of MBAITs at present is that they can be a marker of an underlying *BAP1-*associated germline mutation/cancer syndrome. Individuals with a *BAP1-*germline mutation have an increased risk to develop cutaneous and ocular melanoma and mesothelioma, apart from the risk to develop multiple MBAITs (27).

Management of MBAIT lesions should consist of complete removal of the lesion and advise for genetic counseling to exclude a potential underlying cancer syndrome.

**Figure 3:**
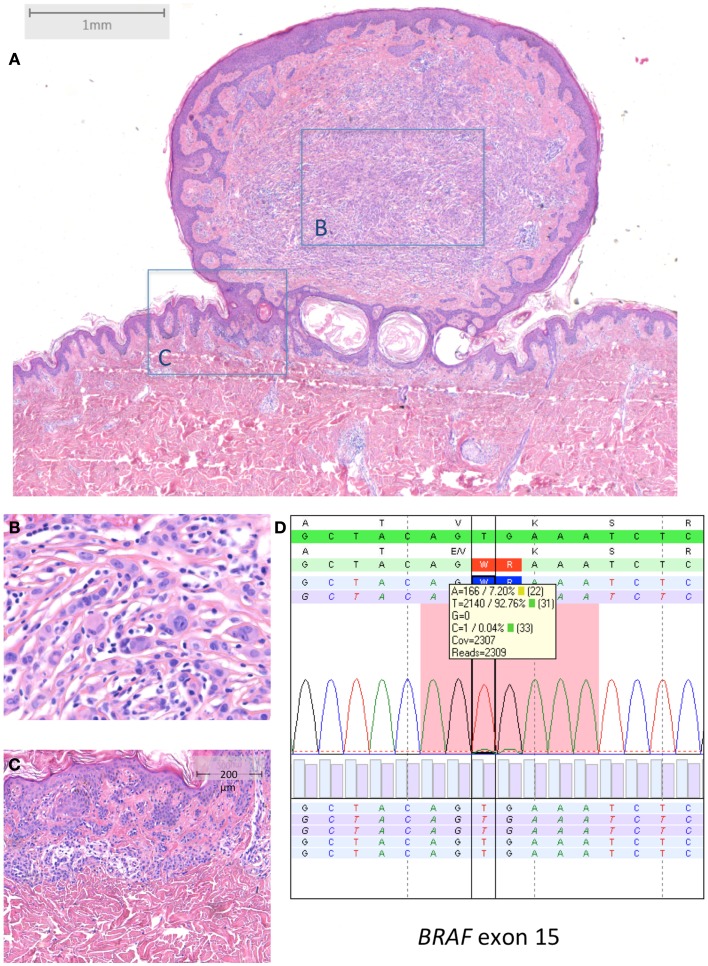
***BRAF*-mutated MBAIT in a 31-year-old female located on the lower back (Courtesy Dr. R. Kornegoor, Gelre Hospitals, Apeldoorn, The Netherlands)**. **(A)** Overview (HE 25×), showing a polypoid lesion above the skin surface. The central part **(B)** (HE 400×) consists of large epithelioid cells with intermingled lymphocytes. The margin **(C)** (HE 100×) shows a common compound nevus. **(D)** Molecular analysis showed a BRAF^V600E^ mutation. In **(D)**, sequence plot of the mutation *BRAF* c.1799_1800TG >AA (p.(Val600Glu) alias p.V600E) with 7% mutant allel (very low load). Note that the *BRAF* RefSeq is NM_004333.4. The sequence plots are visualized as described under Figure 2. Immunohistochemistry demonstrated positive BRAF protein expression, and loss of BAP1 protein expression in the large epithelioid cells (not shown).

**Figure 4:**
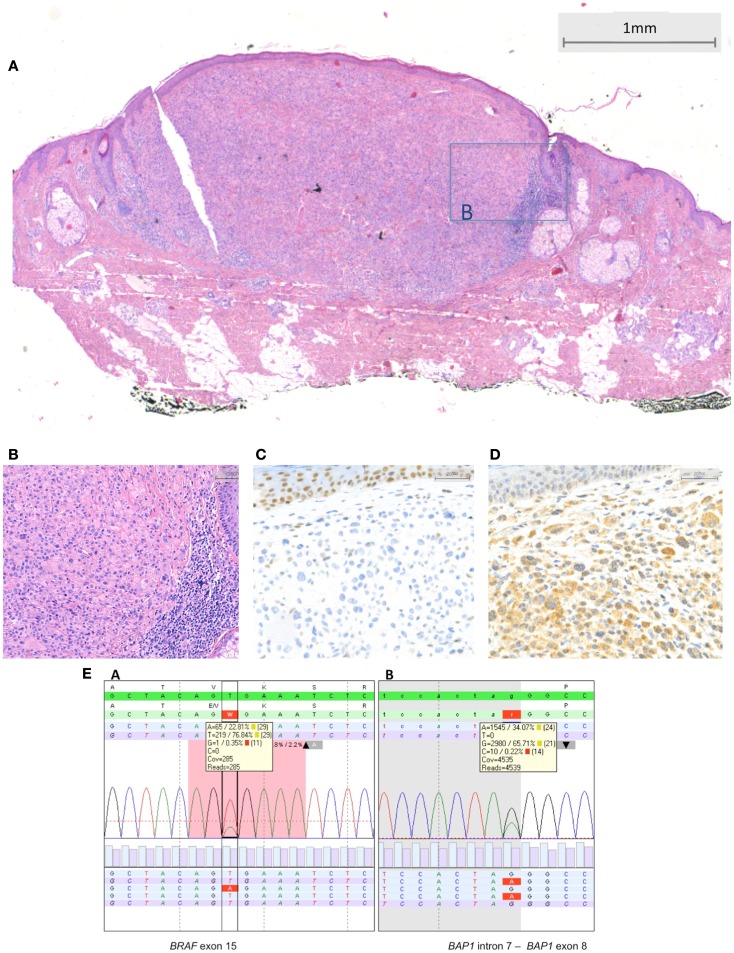
***BRAF*-mutated MBAIT lesion located on the posterior side of the ear in a 55-year-old female (Courtesy Dr. R. Kornegoor, Gelre Hospitals, Apeldoorn, The Netherlands)**. In **(A)**, an overview showing a nodular dermal-based lesion with lymphocytes at the lateral margin. In this case, no common nevus component was present (HE, 25×). **(B)** A higher magnification demonstrates the large epithelioid aspect of the cells (HE, 200×). In **(C)**, absence of BAP1 protein expression is seen in the large epithelioid melanocytes. Nuclei in the keratinocytes in the epidermis stain positive, serving as a positive internal control (400×). In **(D)**, BRAF protein is expressed by the large epithelioid melanocytes (400×). **(E)** Sequence plots of the mutation *BRAF* c.1799T >A (p.(Val600Glu) alias p.V600E) with 23% mutant alleles and of the *BAP1* (RefSeq NM_004656.2) c.581-1G >A splice site mutation with 34% mutant alleles. The sequence plots are visualized as described under Figure 2. In the *BAP1* plot, the intron–exon boundary is represented as gray-white parts.

Typical *BRAF*-mutated MBAIT cases from our own practice are shown in Figures 3 and 4 (Courtesy Dr. R. Kornegoor, Department of Pathology, Gelre Hospitals, Apeldoorn, The Netherlands).

In case of suspicion of a MBAIT lesion, we recommend at least a BRAF and BAP1 immunostaining, but preferentially *BRAF*, *NRAS, HRAS*, and *BAP1* mutation analysis is performed. In case a laboratory cannot perform these, we recommend consultation because of the important implications of a correct diagnosis. Mutation analysis of the *BAP1* gene is difficult, since it is a complex gene and mutations can be present along all of the 17 exons of the gene. A low tumor percentage due to the small size of a lesion or small size of the spitzoid component, or the presence of a lot of TILs, can all hamper BAP1 mutation analysis in this setting.

The third group of spitzoid lesions, those with kinase fusions has only recently been described. Wiesner et al. described the presence of alterations in *ROS1, NTRK1*, *ALK*, *BRAF*, and *RET* in, respectively, 17%, 16%, 10%, 5%, and 3% of spitzoid tumors. These alterations were present along the entire spectrum of the spitzoid tumors (55% in Spitz nevi, 56% in AST, and 39% in spitzoid melanoma), and these alterations therefore seem early events, and are not useful (yet) for differentiating benign from malignant spitzoid lesions. No clear distinct phenotypes were described at this time.

Recently, Busam et al. described 17 cases of Spitz tumors with *ALK* fusion, including 5 Spitz nevi and 12 AST (28). Clinically, these lesions were often polypoid. Histology showed a compound, mostly dermal located lesions with a plexiform growth, and consisting of fusiform melanocytes. In only 2 cases, the lesional cells contained pigment.

All cases showed ALK protein expression. ALK FISH was positive in all cases (using a commercially available break-apart probe, Abbott Molecular, Des Plaines, IL): in 11/17 cases, the fusion partner was tropomyosin 3 (TPM-3), in 6/17 cases, the fusion partner was dynactin 1 (DCTN-1). FISH for copy number alterations did not meet the criteria for melanoma diagnosis in any case. Array CGH revealed no chromosomal gains or losses. In 2 cases, a sentinel node procedure was performed and in both cases showed small nests in the subcapsular sinus. Both patients were alive and well after <1 year and after 4 years of follow-up. At present, the follow-up time and number of cases is too limited to draw definite conclusions about the prognosis of this group of Spitz tumors with *ALK* fusions.

**Figure 5:**
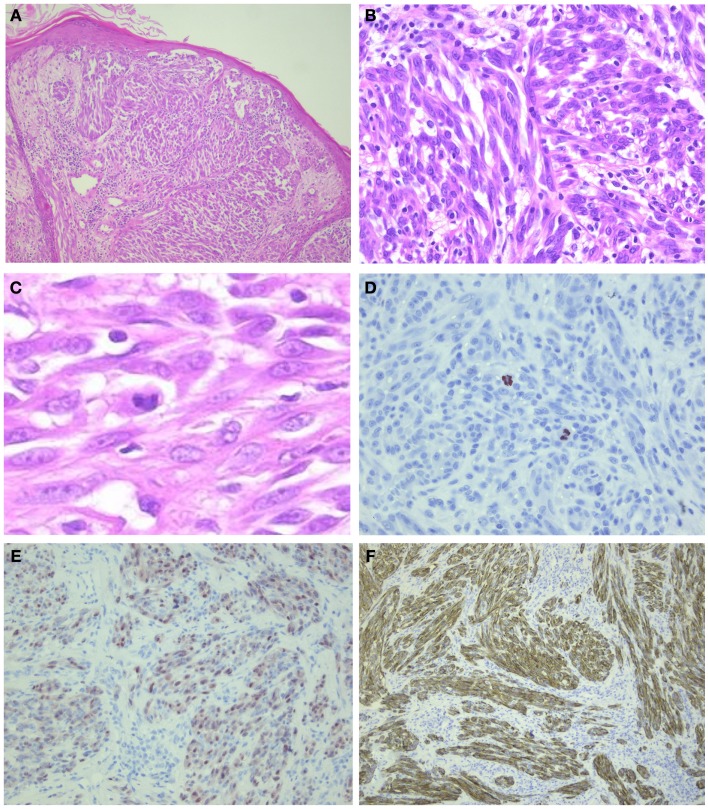
**ALK-positive spitzoid tumor diagnosed as spitzoid tumor of uncertain malignant potential (STUMP)**. This lesion was seen in consultation elsewhere (Courtesy Prof. Dr. J. van den Oord, KU Leuven, Belgium). The lesion was clinically polypoid and presented on the arm of a 4-year-old girl. There were 11 mitoses/1 mm^2^ present. Follow-up data are not available yet. In **(A)**, HE overview (25×); in **(B,C)**, larger magnification (HE 200–400×, respectively) showing spindle-shaped cells and some lymphocytes intermingled; **(D)** PHH3 staining demonstrating mitoses (400×); **(E)** mitf (250×); and **(F)** ALK (250×).

An ALK-positive Spitz tumor is presented in Figure 5 (Courtesy Prof. Dr. J. van den Oord, KU Leuven, Belgium).

The anaplastic lymphoma kinase (*ALK)* gene, located on chromosome 2p23, is a receptor tyrosinase kinase protein and is capable of causing diverse tumor types of different lineages through a variety of molecular mechanisms (29). The most common mechanism of *ALK* activation is a genomic rearrangement involving the *ALK* locus, with a breakpoint in the 3′ end of the *ALK* gene, with the other breakpoint involving a diverse group of genes leading to formation of a fusion oncogene that encodes for a fusion oncoprotein that is able to self-associate (29). Another way of *ALK* activation is by gain-of-function point mutations, but these are less frequent than *ALK* rearrangements. Also, *ALK* amplifications have been described in several tumor types, leading to the presence of multiple copies of the wild-type full-length *ALK* gene. But the way this contributes to tumor development is poorly understood (29).

As mentioned above, in spitzoid tumors at present, only *ALK* rearrangements have been tested and described, and present in upto 19% of cases (16), with *TPM-3* and *DCTN-1* as fusion partners (16, 28). *ALK* rearrangements leading to *TPM3-ALK* fusion have also been reported in anaplastic large cell lymphoma and papillary thyroid carcinoma, and result in a constitutive tyrosinase kinase activity, in this way causing tumor development (29).

Relevance of diagnosis of Spitz tumors with kinase fusions at the moment is mainly for treatment in malignant lesions (e.g., *ALK, RET, ROS 1* alterations can be targeted with kinase inhibitors, such as crizotinib, cabozantinib, and vandetanib).

Table 2 summarizes the characteristic features of the three above-described subtypes of spitzoid melanocytic lesions, including the most common histological findings, and genetic and prognostic features, with clinical relevance of recognition of these subtypes.

**Table 2:** **Subtypes of spitzoid melanocytic tumors with their phenotypical and genotypical characteristics, and relevance of diagnosis**.

Subtypes spitzoid melanocytic tumors	*HRAS*-mutated spitzoid tumors	*BAP 1*-mutated spitzoid tumors (MBAIT)	Spitzoid tumors with kinase fusions
Histological characteristics (phenotype)	Dermal-based lesions	Mostly dermal based	No distinct phenotypes identified yet.
	Wedge shaped	Often polypoid	
	Desmoplasia	Often TILs	
	Often spindle-shaped cells	Epithelioid cells, a common nevus component can be present.	ST with *ALK* fusions recently reported as often polypoid with plexiform growth of fusiform melanocytes (28).
Genetic alterations	*HRAS* mutations or amplification	*BAP1* mutation	Specific fusion of certain genes (*ROS1* 17%, *NTRK1* 16%, *ALK* 10%, *BRAF* 5%, *RET* 3%).
		Often with combined BRAF^V600E^ mutation. Rare cases with combined *NRAS* mutation reported	
	Single 11p gain can be present.	Loss of (a part of) chromosome 3 can be present.	In *ALK-*used STs, no chromosomal gains or losses reported thus far (28).
Relevance of diagnosis	Favorable prognosis (prevention of overdiagnosis of melanoma).	Can be a marker of a BAP1 cancer syndrome. Genetic counseling should be advised.	In case of malignant/metastatic cases targeted treatment is an option (*ALK, ROS*, and *RET* alterations can be targeted by kinase inhibitors like crizotinib, cabozantinib, and vandetanib).
		Behavior not clear yet.	Behavior not clear yet.

*TILs, tumor-infiltrating lymphocytes; ST, Spitz tumor; MBAIT, melanocytic BAP1-associated intradermal tumor*.

### Molecular background of congenital melanocytic nevi (CMN) and neurocutaneous melanosis (NCM)

Congenital melanocytic nevi are pigmented moles present at birth or shortly thereafter (30). They vary in size from small to very large or “giant” and hundreds of CMN can be present in one patient (31). CMN are considered to be a sporadic event with the exception of a few familial cases (32, 33). Especially giant CMN ( >40 cm in size) are associated with an increased risk for cutaneous melanoma (up to 10–15% life-time risk) (34).

Both *NRAS* and *BRAF* mutations have been detected in CMN in a mutually exclusive pattern, and there is a genotype–phenotype correlation between the size of CMN and type of mutation. Especially, large and giant CMN harbor somatic *NRAS* mutations ( >75%) in contrast to medium-sized CMN that are less frequently *NRAS* mutated and especially small CMN and acquired melanocytic nevi that frequently carry BRAF^V600E^ mutations (up to 80% in acquired nevi) (35). Activating BRAF^V600E^ mutations in large CMN are rare (approximately 15%) (6). A few cases of large CMN were shown to harbor a chromosomal translocation involving *BRAF* resulting in gain-of-function, and this could represent an alternative mechanism of BRAF activation in *BRAF*- or *NRAS*-wild-type CMN (36).

Recently, using several highly sensitive techniques, Charbel et al. showed that large and giant CMN contain NRAS^Q61^ mutations (Q61R or Q61K) in up to 94.7% of cases (37). In addition, using whole-exome sequencing, they found no other coding mutations in five large/giant CMN implying that at present *NRAS* mutations are the sole recurrent mutation in these lesions. Somatic *NRAS* mutations, therefore, seem to be sufficient to drive melanocytic proliferations *in utero*. In addition, identical NRAS^Q61^ mutations were recently demonstrated in multiple CMN samples from the same patient (38). This finding suggests that multiple CMN are clonal proliferations caused by a single, postzygotic *NRAS* mutation in a neuro-ectodermal precursor cell rather than *de novo* proliferations arising from different mutations.

Kinsler et al. recently proposed the term “CMN syndrome” as they observed that patients with CMN often have characteristic facial features (such as wide or prominent forehead and apparent hypertelorism) (39). The osteocartilageneous structures of the face are neuro-ectodermal in origin and can be affected by mutations in components of the RAS/RAF/MEK/ERK pathway in patients with germline RASopathies who have characteristic facial features (40). Kinsler et al. hypothesized that the occurrence of a postzygotic, somatic *NRAS* mutation in early neuro-ectodermal precursors might be responsible for the characteristic facial features in patients with CMN as well (38). In addition, as they observed a high prevalence of red hair in families of children with CMN, a germline predisposition for the development of the “CMN syndrome” was hypothesized. Indeed, they showed that certain germline allele variants of the melanocortin-1-receptor (MC1R), known to be responsible for the red hair/fair skin/freckling phenotype, are associated with the presence of CMN (as well as with more extensive CMN) (41).

Congenital melanocytic nevi can be associated with a spectrum of neurological abnormalities, including malformations (for instance, Dandy–Walker malformation), communicating hydrocephalus, arachnoid cysts, CNS tumors (for instance, astrocytoma and choroid plexus papilloma) and also melanotic abnormalities (38). The latter is called neurocutaneous melanosis (NCM) and refers to the presence of large of multiple CMN in association with melanin depositions in the brain parenchym (visible on T1-weighted MRI) or melanocytic tumors like leptomeningeal melanocytosis or melanoma (42). Leptomeningeal melanocytosis consists of a diffuse proliferation of histological benign appearing melanocytes in the leptomeninges, without CNS invasion, and carries a poor prognosis once symptomatic (42).

Recently, it was shown that postzygotic, somatic *NRAS* mutations contribute to the pathogenesis of NCM. Identical, somatic NRAS^Q61^ mutations were detected in multiple CMN as well as in the CNS melanocytic tumor in the same NCM patients (43). Kinsler et al. also detected a somatic NRAS^Q61^ mutation in three non-melanocytic CNS tumors occurring in patients with CMN (including choroid plexus papilloma, neurocristic hamartoma, and meningioma) (38). The presence of identical, somatic *NRAS*Q61 mutations in both CMN and in CNS melanocytic (and non-melanocytic) neoplasms in the same patients suggests that these mutations occur in the developing neuro-ectoderm early during embryogenesis. In fact, this pathogenetic mechanism fits the spectrum of mosaic RASopathies that are characterized by postzygotic mutations resulting in the presence of at least two genetically distinct cell populations in the same organism (44). For instance, postzygotic, somatic *RAS* mutations (*HRAS, KRAS*) were recently shown to be present in distinct lesions of the keratinocytic epidermal nevus syndrome, a mosaic RASopathy characterized by the presence of epidermal nevi in association with extra-cutaneous abnormalities (CNS, ocular, skeletal, cardiovascular, and genitourinary system) (44). The phenotype in the spectrum of mosaic RASopathies is most likely determined by type of mutation, the timing of the mutation, and affected cell type (44, 45).

A mouse model has demonstrated a role for postzygotic, early embryonic *NRAS* mutations in the pathogenesis of NCM. By using the Cre-loxP technology, Pedersen et al. showed that expression of oncogenic *NRAS*^G12D^ in melanocytes early during embryogenesis resulted in a mouse phenotype strongly resembling NCM in human beings (43) As *NRAS* mutations occur in benign lesions such as CMN, they are in itself insufficient for malignant transformation of melanocytes, and better insight in the genetic aberrations eventually leading to melanoma is needed. *NRAS* mutations are a therapeutic target for treatment with MEK inhibitors. In a Phase II trial, treatment with MEK162 was shown to have effect in some patients with advanced *NRAS*-mutated melanoma and a Phase III trial is ongoing (46). For patients with *NRAS*-mutated CNS melanocytic tumors, treatment with MEK162 might be of benefit as well (47).

### Molecular diagnostics in the differential diagnosis of melanoma (metastasis)

Melanomas are known for their wide range of cytomorphologic features and architectural patterns and may mimic various non-melanocytic tumors [reviewed by Banerjee et al. (48, 49)]. In most cases, a diagnosis can be rendered by careful examination of the histomorphology and by sometimes adding immunohistochemical stains. In some cases, however, especially in recurrences and metastases, melanomas can show an aberrant immunophenotype with loss of lineage-specific markers. In part of these cases, molecular analysis of genes in the MAPK pathway and *CDKN2A* (7) mutation analysis can be useful in the differential diagnosis. In addition, molecular analysis can be of help in determining the site of the primary melanoma in case of metastasis with an unknown primary since melanomas from different locations have different mutation types (see Table 1). Furthermore, molecular analysis can help to discriminate between a melanoma metastasis and a second primary melanoma.

One of the lesions that can be hard to distinguish from melanoma is clear cell sarcoma (CCS) as they share histopathological features and cannot be distinguished by immunohistochemistry (50). Classically, CCS is a deep soft tissue tumor associated with tendons and aponeuroses (51), but it can also present as a cutaneous lesion (52), and then has to be differentiated from metastatic or primary nodular melanoma. While most cutaneous melanomas harbor a *BRAF* or *NRAS* mutation (see Table 1), CCS in approximately 75% have a t(12;22)(a13;q12) or less commonly a t(2;22)(q34;q12) translocation leading to the *EWSR1/ATF1* or *EWSR1/CREB1* fusion transcripts (53, 54). Yang et al. (55) performed *BRAF* and *NRAS* mutation analysis, as well as FISH analysis for the *EWST1/ATF1* fusion gene in 31 melanoma cases and 16 CCSs. They found the translocation in 78.6% of the CCSs but in none of the melanomas, whereas *BRAF* and *NRAS* mutations were present in, respectively, 51.6 and 12.9% of the melanomas and not in any of the CCSs. Hantschke et al. (52) described 12 cases of cutaneous CCS in which FISH analysis for the t(12;22)(a13;q12) translocation contributed in the differential diagnosis with melanoma. This implicates that this type of analysis can be of great aid in the differential diagnosis between CCS and melanoma.

Recently, we described two cases in which mutation analysis lead to the correct diagnosis of (dedifferentiated) metastatic melanoma (56). Both patients had a history of melanoma and presented several years after their primary diagnosis with a lesion histologically and immunohistochemically different from the primary melanoma and mimicking a solitary fibrous tumor (SFT). Using *BRAF* and *NRAS* mutation analysis, it could be proven that both lesions were melanoma metastases instead of SFT. Many other soft tissue tumors can mimick melanoma or melanoma metastasis, such as dermatofibrosarcoma protuberans, malignant peripheral nerve sheath tumors (MPNST) (57), and sarcomas (48, 58–60).

**Figure 6:**
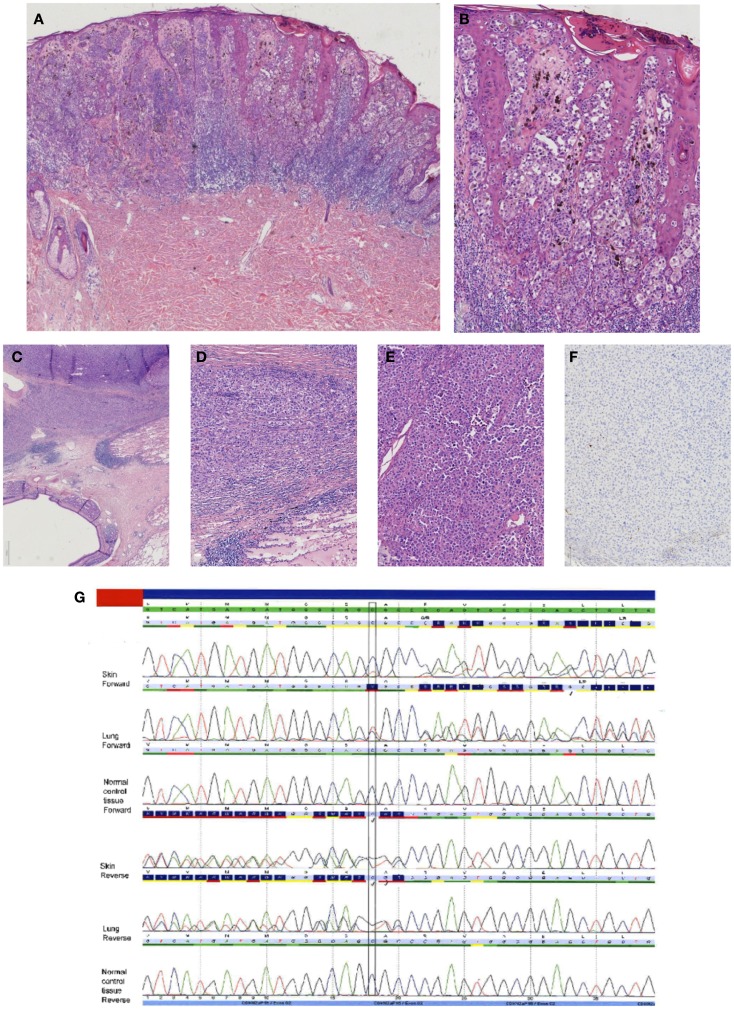
**Case of a superficial spreading melanoma of the back with lung metastasis 16 years later in a female patient**. In **(A)**, an overview of the primary cutaneous melanoma showing centrally an asymmetrical melanocytic lesion (HE, 25×). In **(B)**, a more detailed picture showing clear atypia with loss of maturation and ascension in the epidermis (HE, 100×). In **(C–E)**, the lung lesion at different magnifications [HE C 25×, **(D,E)** 100×] showing a nodular proliferation of atypical partly plasmacytoid cells resembling the primary cutaneous melanoma. Immunohistochemical S100 stain, however, was negative [**(F)** S100 immunostaining, 100×]. Internal and external controls were positive. **(G)** Sequence plots demonstrated an identical c.168_170delinsTG(p.(Arg58fs)) in the *CDKN2A* gene in both the skin melanoma and the lung melanoma, which was absent in normal tissue of the patient. This confirmed that the lung tumor was a metastasis of the previous melanoma. The sequence plots are visualized as described under Figure 2.

Although *CDKN2A* mutation frequency in sporadic melanomas as well as consistency in melanoma metastasis are reported to be low [reported *CDKN2A* mutation frequency in upto 25% of primary melanomas, 0–14% in melanoma metasases, with a 31% consistency being reported between a primary and the metastasis (61, 62)], *CDKN2A* mutation analysis can be an alternative way to confirm a diagnosis of metastatic melanoma by showing a clonal relationship between a primary melanoma and a metastasis [after exclusion of a *CDKN2A* germline mutation (63)], as is illustrated by a recent case from our own practice. A 36-year-old woman with a history of a superficial spreading melanoma of the back 16 years earlier, presented with a large lung mass, which was thought to be a non-small cell lesion, probably squamous cell carcinoma based on fine needle aspiration (EUS-FNA); melanoma markers HMB45 and MART were negative, while the squamous cell marker – p63 – was weakly positive. Evaluation of the pneumonectomy specimen, however, led to a differential diagnosis with metastatic melanoma, since immunohistochemical stainings on the complete tumor now available for histological evaluation showed in part features consistent with melanocytic differentiation. Vimentin, tyrosinase, and mitf were weakly positive; however, S100 and SOX10 were both negative. Therefore, additional *BRAF* and *CDKN2A* mutation analysis was performed. Identical mutations in both genes were present in the primary cutaneous melanoma and the lung mass – in absence of a germline *CDKN2A* mutation – confirming that the lung mass was a late metastasis of the cutaneous melanoma. This case is illustrated in Figure 6.

In a patient with a history of melanoma, it can occasionally be difficult to differentiate between a second primary cutaneous melanoma and a melanoma metastasis based on histomorphology, especially when there is epidermotropism (64). If the first melanoma was not a cutaneous melanoma, the problem can often be solved by mutation analysis since different types of melanomas have mutations in different genes of the MAPK pathway as mentioned in Table 1. If the primary melanoma was a cutaneous melanoma, mutation analysis of *NRAS* and *BRAF* can be of help, but since these mutations are hotspot mutations, it is of limited use. Usage is additionally hampered by frequent occurrence of heterogeneity in melanoma with respect to NRAS and *BRAF* status, leading to a substantial discordance in mutation status in these genes in a primary compared to the metastases (61, 65).

*CDKN2A* mutation analysis can sometimes be helpful as we have published before (66), and is illustrated by the case above, to differentiate between a new primary and a melanoma metastasis. The advantage of using *CDKN2A* mutations for clonality is that *CDKN2A* mutations are unique mutations instead of hot spot mutations. Although *CDKN2A* mutation frequency in sporadic melanomas is low, *CDKN2A* mutation analysis to our opinion is worth trying, as there is a substantial difference in the prognosis between a second primary melanoma and a melanoma metastasis.

## Part 2: Molecular Pathology in Melanoma Treatment

Metastatic melanoma treatment got a great impulse after the discovery that the (hot spot) mutations in genes involved in the MAPK pathway in melanoma, as well as in *GNAQ* and *GNA11*, which proved targetable on the protein level by specific inhibitors. *GNAQ* and *GNA11* mutations are mainly present in uveal and primary brain melanomas, and combinations of MEK inhibitors with either PI3K or mTOR inhibitors have shown efficacy in *GNAQ-* and *GNA11*-mutant melanomas (67, 68). A simplified scheme of the most important pathways involved in melanoma pathogenesis, and points of action of specific inhibitors are depicted in Figure 1.

Melanomas from distinct locations have been shown to contain different mutation types and frequencies (5, 11, 12). An overview of the most frequent mutations in the distinct melanoma subtypes and their frequencies, as well as the different therapeutic options, are depicted in Table 1.

The most frequently tested genes at present in melanoma are *BRAF* and *NRAS*, because these mutations are most prevalent in melanoma, especially in those arising from the skin. Testing for targeted treatment in melanoma should be guided by the localization of the primary tumor. Nowadays, this issue becomes less important because several molecular labs use platforms that detect various regions from multiple genes within one test.

In case of molecular testing for targeted therapy in metastatic melanoma, there are several important issues.

Molecular testing for therapy requires a close collaboration between clinician, pathologist, and molecular biologist. Testing for treatment should only be performed when targeted therapy is considered as a therapeutic option. This can be judged best by the clinician. In most cases, testing is only indicated in inoperable stage 3 or stage 4 melanoma patients.

Tests need to be performed in an accredited molecular laboratory that guarantees that the laboratory techniques and processes are performed standardized and yield high-quality results, which imply that only validated tests are used.

The role of the pathologist is to confirm the diagnosis of melanoma metastasis, to check representativity of the tumor tissue to be examined, to indicate the area for macrodissection (if needed) by the technician for DNA extraction, to indicate possible tumor heterogeneity, and to estimate the neoplastic cell percentage within the tested sample.

The knowledge and the expertise of trained molecular biologists are used to come to optimal test results and adequate interpretation and reporting of molecular test results. The molecular report should contain information on the specific tissue block tested, the percentage of neoplastic cells, the type of molecular test used, and the sensitivity of the test, the type of genes, and exons or mutations thereof which are tested, and an accurate description of the mutation present according to the Human Genome Variation Society nomenclature. The molecular biologist, together with the pathologist, is responsible for proper integration and interpretation of the molecular results in the pathology report.

The molecular test is preferentially performed on a recent metastasis. First, reason for this is confirmation of the diagnosis of melanoma metastasis. Second, there is considerable tumor heterogeneity with a reported discrepancy by Colombino et al. of 7.5–29% in the *BRAF* and *NRAS* mutation status of the metastatic melanoma when compared to the primary (61, 65). Concordance seems dependent on the location of the metastases and is highest for visceral (92.5%) and nodal metastases (91%), but relatively low for brain (79%) and skin metastases (71%). Especially, in the latter, testing should be performed on the metastasis.

Saint-Jean et al. (69) also reported discordant *BRAF* results. They performed multiple tests in a subgroup of 74 patients: in 10/74 (13.5%) of these patients, *BRAF* status was discordant between distinct samples of a patient. In two patients, the discordance was present between the primary and a metastasis. In six patients, the discordance was present between two distinct metastases. The authors state that without repeated testing, five patients would unjustly have been excluded from treatment with the *BRAF* inhibitor, vemurafenib. They advocate to test other samples in case no *BRAF* mutation has been detected. We can agree with this especially in selected cases, when the primary location is likely to be associated with a *BRAF* mutation and when no mutation in other targetable genes (like *NRAS* or *KIT*) have been identified.

A molecular test should be able to detect all relevant and targetable mutations in a gene. The most frequently tested gene for melanoma treatment at present is *BRAF*.

The most frequent *BRAF* mutation is a mononucleotide point mutation in codon 600 (CTG) of exon 15, c.1799T >A (p.(Val600Glu)), in which the valine (V) of codon 600 is replaced by glutamine (E). This mutation is, therefore, known as the BRAF^V600E^ mutation and is present in about 70–75% of all *BRAF*-mutated melanomas (11, 70, 71). In a recent study in which 1112 primary and metastatic melanomas from different locations were analyzed for *BRAF* mutations (774 skin melanomas, 111 acral melanomas, 26 mucosal melanomas, 23 uveal melanomas, 1 leptomeningeal melanoma, and 177 metastases), 44.9% of the cases harbored a *BRAF* mutation: in 75.4% of the cases, mutations were BRAF^V600E^ either deriving from the c.1799T >A or from a c.1799 1800delinsAA mutation. Of the remaining BRAF^non-V600E^ cases (24.6%), the most frequently seen mutation was the BRAF^V600K^ (17.2%); BRAF^V600R^ or BRAF^V600D^ mutated cases were found in low percentages (4.6%). *BRAF* exon 11 mutations were also observed in a low percentage (0.4%).

There are several studies that have reported that all melanomas with BRAF codon 600 mutations are sensitive to *BRAF* inhibitors, such as vemurafenib (Zelboraf^®^, Roche Molecular Systems Inc.) and dabrafenib (GSK2118436) (72, 73). This implicates that a *BRAF* test needs to be able to detect not only the BRAFc.1799T >A(p.(Val600Glu)/BRAF^V600E^ mutation but the other codon 600 mutations as well.

A test must be able to detect *BRAF* mutations that have been reported to be insensitive to *BRAF*-inhibitor treatment, like the kinase-dead mutation BRAF^D594^ (74, 75).

The molecular test for therapy should be performed within a short turnaround time since mostly this kind of targeted therapy will be given in rapidly progressive metastatic melanoma patients. A turnaround time of 5 working days is feasible within our hands.

At present, we perform NGS-based mutation testing using Ion Torrent Personal Genome Machine (IT-PGM) from Life Technologies for analysis of gene-panels for diagnostic purposes.

At the moment, there is a tendency toward testing with NGS methods for targeted treatment in diverse cancer types. The sensitivity of NGS is higher than Sanger sequencing (detection of 2–10% versus 15–25% allele frequency). Moreover, the amount of DNA that is needed for the analysis of gene panels is very low, only 10 ng for *all* amplicons for instance when using the IT-PGM from Life Technologies versus 10 ng needed *per* amplicon for Sanger sequencing.

The turnaround time and costs of NGS methods can be competitive with respect to low throughput technologies in centers that have sufficient numbers of samples.

For diagnostic requests in melanoma in our department, a custom-designed gene panel for amplicon-sequencing of *BRAF* (NM_004333.4) exon 15, *NRAS* (NM_002524.4) exon 2 and 3, *HRAS* (NM_005343.2) exon 2 and 3, *AKT1* (NM_005163.2) exon 3, *GNAQ* (NM_002072.2) exon 4 and 5, *GNA11* (NM_002067.2) exon 4 and 5, *KIT* NM_000222.2) exon 8, 9, 11, 13, and 14, and *PDGFRA* (NM_006206.4) exon 12, 14, and 18 is performed.

The use of small, dedicated gene panels and efficient loading of the chips for IT-PGM also significantly reduce costs per case.

The major benefit of (targeted) NGS is that it uncovers all kinds of mutations in selected genomic regions instead of only mutations at predefined positions.

## Conflict of Interest Statement

The authors declare that the research was conducted in the absence of any commercial or financial relationships that could be construed as a potential conflict of interest.
